# Pasireotide-LAR and pegvisomant combination in resistant acromegaly: a case series of predominantly mammosomatotroph adenomas

**DOI:** 10.1007/s11102-026-01750-1

**Published:** 2026-09-23

**Authors:** Edward Mignone, Elena V. Varlamov, Rinki Pandya, Melanie Hakar, Maria Fleseriu

**Affiliations:** 1 School of Medicine, Adelaide University, Adelaide, SA Australia; 2 Department of Neurological Surgery, Oregon Health & Science University, Portland, OR USA; 3 Pituitary Center, Oregon Health & Science University, Portland, OR USA; 4 Department of Medicine, Division of Endocrinology, Diabetes and Clinical Nutrition, Oregon Health & Science University, Portland, OR USA; 5 Department of Pathology and Laboratory Medicine, Oregon Health & Science University, Portland, OR USA

**Keywords:** Acromegaly, Pasireotide, Pegvisomant, Treatment-resistant, Mammosommatotroph adenomas

## Abstract

**Purpose:**

Long-term real-world data on concurrent pasireotide-LAR (PAS) and pegvisomant (PEGV) use in treatment-resistant acromegaly remain limited, especially for mammosomatotroph adenomas in younger adults. We report outcomes of six patients treated with this combination at a single tertiary pituitary center.

**Methods:**

IRB-approved retrospective case series. Baseline and follow-up biochemical, radiological, histopathological, treatment and safety outcomes were captured.

**Results:**

Six patients (5 females, 1 male; age 15 to 44 years) with somatotroph (*n* = 2) or mammosomatotroph (*n* = 4) adenomas, all macroadenomas, received PAS (20 to 60 mg every 28 days) plus PEGV (60 to 280 mg/week) over a median of 3.3 years (range 0.5 to 5.9) after lack of disease control post-surgery despite multiple lines of medical therapy. All six maintained tumor stability and achieved normal IGF-1. One had biochemical escape after 9 months of biochemical control requiring a 2nd surgery and radiosurgery. One patient discontinued PEGV after 3 years due to injection site pain, 3 patients developed hyperglycemia in the pre-diabetes range; no hepatotoxicity, acute symptomatic biliary disease, or arrhythmias occurred.

**Conclusions:**

In this series of predominantly mammosomatotroph adenomas in younger adults, combination PAS plus PEGV offers potential durable biochemical control, tumor stability and PEGV dose reduction in treatment-resistant acromegaly. Glucose monitoring during treatment is essential as concomitant use of PEGV does not appear to mitigate PAS-induced glycemic deterioration. Larger prospective studies are needed to better define predictors of response.

## Introduction

Biochemical control of acromegaly remains challenging in patients with invasive macroadenomas that are resistant to somatostatin receptor ligands, e.g. octreotide LAR, lanreotide, paltusotine [1]. Tumor biology factors including sparsely granulated histology, T2 hyperintensity on MRI, and low somatostatin receptor subtype 2 (SSTR2) expression may predict this resistance [2, 3]. Pasireotide-LAR (PAS) is a multireceptor ligand with a higher affinity for SSTR5 and improved efficacy in this setting, resulting in biochemical control in an additional 25% of patients [4]. It's combination with the growth hormone (GH) receptor antagonist pegvisomant (PEGV) provides a complementary mechanism by targeting both GH secretion and peripheral GH action [5]. Due to limited real-world data, this combination is listed as a discretionary third-line recommendation in the most recent international acromegaly consensus [2]. We report a case series of six patients treated with this combination at a single tertiary Pituitary Center, with particular attention to biochemical and radiological outcomes in a predominantly mammosomatotroph cohort.

## Methods

This IRB-approved retrospective review identified six patients with acromegaly treated with concurrent PAS and PEGV due to inadequate biochemical control despite surgery and multiple lines of prior medical therapy. IGF-1 trends, pathology, MRI adenoma response, and safety with prolonged treatment were assessed.

## Case series

Six patients (five females, one male; age range 15–44 years at diagnosis) with macroadenomas received PAS and PEGV (PAS-PEGV) combination following inadequate biochemical control after surgery. Full baseline characteristics, treatment details, and outcomes are summarized in Table 1.

**Table 1:** Baseline characteristics, treatment details, and outcomes for six patients with treatment-resistant acromegaly treated with concurrent pasireotide-LAR and pegvisomant. Prior therapies are listed in chronological sequence

	Patient 1	Patient 2	Patient 3	Patient 4	Patient 5	Patient 6
*Baseline Characteristics*						
Age at diagnosis / Sex	35, F	44, F	33, F	15, F	21, M	29, F
Adenoma subtype*	Mammosomatotroph	Mammosomatotroph	Somatotroph	Mammosomatotroph	Mammosomatotroph	Somatotroph
SSTR2 expression**	Negative	Positive	Not reported	Positive	Positive	Negative
Ki-67	2–4%	8–10%	2%	< 1%	1–2%	5%
Tumor size (mm)	19 × 16 × 14	39 × 37 × 28	22 × 17	29 × 24 × 16	32 × 26 × 24	17 × 15
Knosp grade	1	3	1	3	2	1
Baseline IGF-1 (ng/mL)	963 (81–278)	> 1,200 (69–253)	> 1,200 (59–279)	1,386 (218–659)	694 (109–353)	1051 (117–321)
Prior therapies (in sequence)	TSS ➜ PAS ➜ PAS + PEGV	TSS ➜ Lanreotide + CAB ➜ 2nd TSS ➜ PAS ➜ PAS + PEGV ➜ PAS	Octreotide- LAR ➜ TSS ➜ PEGV ➜ Octreotide- LAR + PEGV ➜ PAS + PEGV	TSS ➜ _PAS ➜ PAS + PEGV ➜ PAS + PEGV + CAB ➜ 2nd TSS ➜ GKRS ➜ PAS + PEGV + CAB	TSS ➜ Octreotide-LAR ➜ PAS + PEGV	TSS ➜ 2nd TSS ➜ Lanreotide ➜ Lanreotide + PEGV ➜ PAS + PEGV ➜ 3rd TSS
*Combination Therapy (maximum doses)*						
Pasireotide-LAR dose (mg/28 days)	40	60	40	60	40	40
Pegvisomant dose (mg/week)	60	140	210	140	90	280
Duration on combination (years)	5.9	3	4.9	3.5	2.8	0.5
*Outcomes*						
IGF-1 nadir on PAS + PEGV (ng/ mL)	185 (71–258)	179 (52–328)	100 (83–280)	334 (162–458)	115 (109–353)	279 (52–326)
IGF-1 at last follow-up (ng/mL)	220 (71–258)	350 (50–317)	141 (78–274)	479 (117–436)	312 (109–353)	279 (52–326)
Tumor response on MRI	SD	SD, reduction of the cystic portion	SD	SD	SD	SD
Hyperglycemia	No	Yes – HbA1c 6.1%	Yes – HbA1c 6.2%	Yes – HbA1c 6.1%	No	No
Vertebral fracture incident	None	Not recorded	Not recorded	Not recorded	None	None
Other adverse effects	None	Injection site pain, GI disturbance	None	None	Flushing, injection site pain, GI disturbance	None

*CAB* cabergoline, *F* female, *GH* growth hormone, *GI* gastrointestinal, *GKRS* gamma-knife radiosurgery, *HbA1c* glycated hemoglobin, *Ki-67* proliferative index, *M* male, *PAS* pasireotide long-acting release, *PEGV* pegvisomant, *PRL* prolactin, *SD* stable disease, *SSTR2* somatostatin receptor subtype 2, *TSS* transsphenoidal surgery, *ULN* upper limit of normal

*Mammosomatotroph defined as GH and PRL staining in the same cell [6]

**Negative SSTR2 defined as < 10% expression in tumor tissue

### Patient 1

**Fig. 1:**
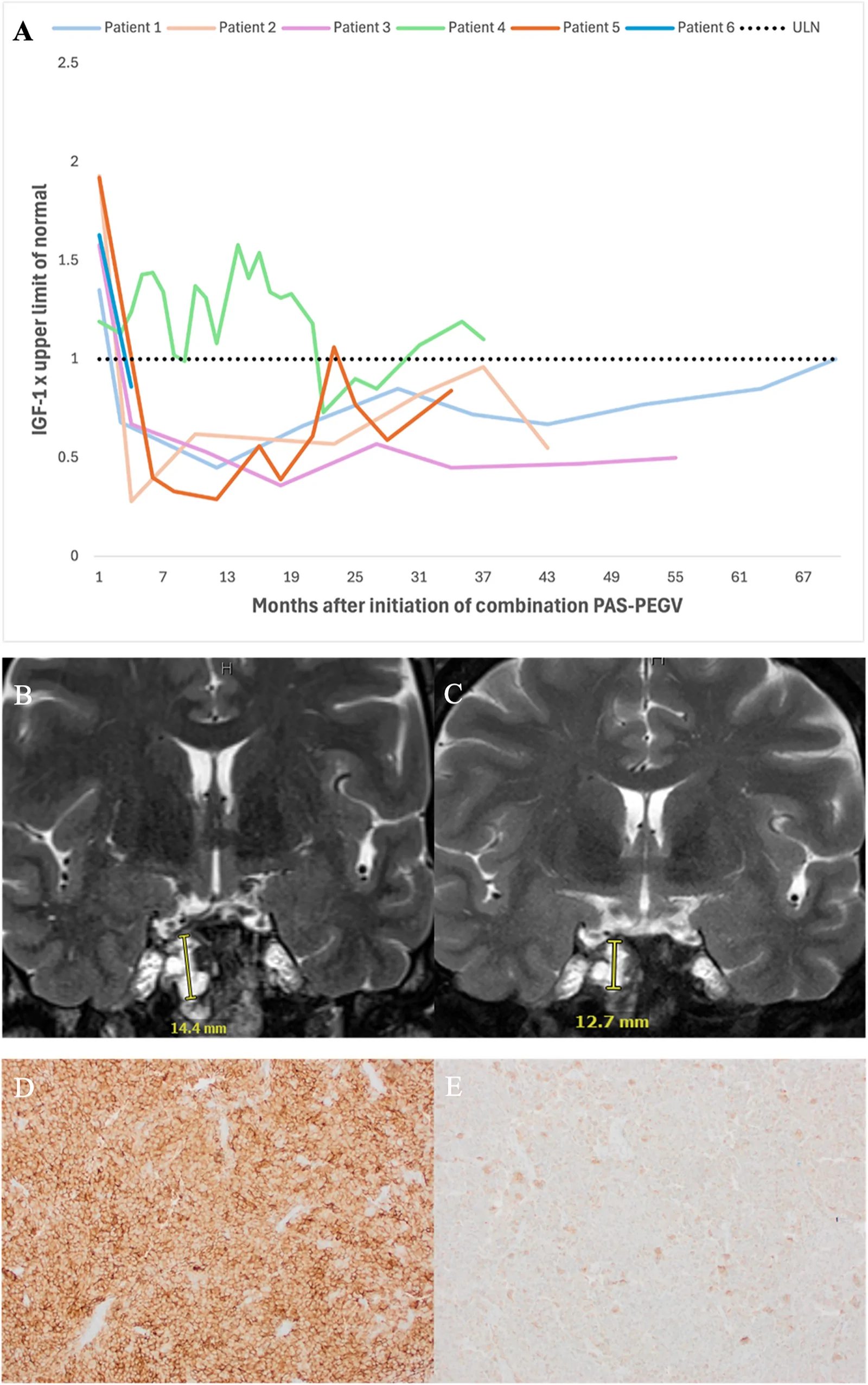
Graphical representation of IGF-1 trajectory for each patient during combination pasireotide-pegvisomant (PAS-PEGV) therapy (**A**). IGF-1 values are expressed as a multiple of the age-adjusted upper limit of normal (ULN; horizontal reference line) and plotted against time in months from initiation of combination therapy. T2-weighted coronal MRI of Patient 2 at baseline (**B**) and after 24 months of PAS-PEGV therapy, but prior to radiosurgery (**C**), showing reduction in cystic component dimension from 14.4 mm to 12.7 mm. Histopathology of Patient 4's adenoma (original magnification x100) showing strong, diffuse SSTR2 immunopositivity (**D**) and prolactin immunopositivity in the majority of tumor cells (**E**)

A 35-year-old woman with a SSTR2-negative mammosomatotroph macroadenoma had failure of biochemical control on PAS monotherapy following transsphenoidal surgery (TSS). IGF-1 normalized after combination therapy was initiated at PAS 40 mg monthly plus PEGV 60 mg/week and has remained within the normal range over 5.9 years (Fig. 1). No hyperglycemia or adverse effects occurred.

### Patient 2

A 44-year-old woman with an invasive mammosomatotroph macroadenoma requiring two TSS and subsequent panhypopituitarism achieved only partial biochemical control with lanreotide plus cabergoline. Combination PAS 60 mg monthly plus PEGV 140 mg/week achieved an IGF-1 nadir of 179 ng/mL (52–328 ng/mL). PEGV was discontinued after 3 years due to injection site pain and gastrointestinal side effects. IGF-1 was 350 ng/mL (50–317 ng/mL) at last follow-up on PAS 60 mg monthly monotherapy.

### Patient 3

A 33-year-old woman with a somatotroph macroadenoma commenced PAS 40 mg monthly plus PEGV 150 mg/week following TSS and sequential PEGV plus octreotide-LAR that failed to achieve biochemical control. IGF-1 normalized within 3 months and has been maintained over 4.9 years. Mild hyperglycemia developed, improved on tirzepatide utilized for weight management, and did not necessitate discontinuation. No other adverse effects occurred.

### Patient 4

A 15-year-old girl with an invasive mammosomatotroph macroadenoma with no pathogenic germline variants underwent surgical debulking followed by PAS monotherapy. PEGV was added and progressively up titrated over 12 months to achieve IGF-1 normalization with PAS 60 mg monthly and PEGV 140 mg/week. Biochemical escape occurred after 9 months, unresponsive to the addition of cabergoline 1 mg weekly, culminating in repeat surgery (craniotomy) and gamma-knife radiosurgery (GKRS). No tumor growth occurred on PAS-PEGV therapy. Pasireotide-induced hyperglycemia required addition of metformin. IGF-1 was 479 ng/mL (117–436 ng/mL) at last follow-up on PAS 60 mg monthly, PEGV 140 mg/week plus cabergoline 1 mg/week.

### Patient 5

A 21-year-old man with a mammosomatotroph adenoma without pathogenic germline variants remained uncontrolled on maximum-dose octreotide-LAR plus PEGV post TSS. Following uptitration of PAS to 60 mg monthly and PEGV to 90 mg/week, IGF-1 normalized and has been sustained at last follow up. Injection site pain, loose stools and flushing occurred but did not require treatment discontinuation.

### Patient 6

A 29-year-old female with a sparsely granulated somatotroph macroadenoma presented with adenoma growth and lack of biochemical remission despite two TSS, lanreotide monotherapy and combination lanreotide-PEGV (IGF-1 430 ng/mL, 73–263). She underwent a 3rd TSS with preoperative combination PAS-PEGV and was able to achieve rapid biochemical normalization (IGF-1 279 ng/mL, 52–326) within 3 months.

## Discussion

Patients with acromegaly with disease resistant to octreotide, lanreotide or paltusotine could benefit from either combination with pegvisomant or switching to either pegvisomant or pasireotide [1, 7]. Large, more aggressive adenomas may require more potent regimens, thus a combination of PAS-PEGV has been suggested as third-line therapy, yet real-world outcome data remain very limited, especially for mammosomatotroph adenomas in younger patients [2]. We report outcomes over a median follow-up of 3.3 years in six patients commenced on PAS-PEGV due to uncontrolled disease despite prior surgery and medical therapy, representing among the longest follow-up reported for this combination, particularly for mammosomatotroph adenomas.

### Biochemical response

All six patients achieved normal IGF-1 nadir levels on PAS-PEGV combination therapy, with a time to normalization of 12 to 36 weeks, consistent with response rates of 67 to 100% reported across prior studies [8–11]. One patient escaped biochemical control at nine months after achieving normal IGF-1 without evidence of tumor growth, ultimately requiring repeat surgery and GKRS. The PAPE studies, the largest prospective data available, reported IGF-1 normalization in 73.8% and 77% of patients respectively at 24 and 48 weeks. These cohorts were selected on the basis of prior biochemical control on octreotide/lanreotide-PEGV, representing a less refractory population than ours [8, 9]. A retrospective cohort with PAS-PEGV initiation due to treatment resistance reported IGF-1 normalization in all six patients, though follow-up was shorter [10]. Taken together, these findings suggest that combination PAS-PEGV can achieve long-term biochemical control in severely refractory disease and may warrant consideration earlier in the treatment course, rather than reserved only as third-line therapy.

### Radiological response

Tumor stability was maintained in all patients, contributing novel real-life radiological data to this combination [8, 9, 11, 12]. Among prior case series, Chiloiro et al. 2019 reported no tumor regrowth in all six patients, with one demonstrating MRI-confirmed shrinkage [10], Ciresi et al. reported tumor stability during the combination phase following prior shrinkage on PAS monotherapy [13], and Giambò et al. confirmed stable residual adenoma at eleven months of combination therapy [14], all broadly consistent with our findings. Pasireotide’s superior anti-tumor effect vs. octreotide and lanreotide may reflect anti-proliferative mechanisms beyond SSTR2, due to progressive T2-signal hyperintensity consistent with cystic degeneration in patients on pasireotide-based therapy [15], which can contribute to the dose decrease seen over time and also tumor shrinkage with apoptosis in some patients treated with PAS [16].

### Safety profile

The combination was generally well tolerated. Three of six patients (50%) had deterioration in glucose metabolism within a pre-diabetes range, but no treatment discontinuations occurred. This rate is higher than the 16.7% worsening reported by Chiloiro et al. 2021 [11], but is below the lower end of the reported 60–90% incidence of hyperglycemia for patients treated with PAS [17]. This suggests that the PEGV addition may have conferred some marginal glycemic protection in our cohort, however, younger age of our patients and the independent effect of GH/IGF-1 normalization on glucose metabolism may also have contributed to this observation, since effective biochemical control of acromegaly by any means reduces GH-mediated peripheral insulin resistance [2]. Despite previous suggestions that PEGV may improve glucose homeostasis for individuals on PAS [11], our findings are consistent with the PAPE study that PEGV’s insulin-sensitizing effect does not significantly offset PAS’s direct suppression of insulin secretion and incretin release via pancreatic SSTR5 [18, 19]. No hepatotoxicity, acute symptomatic biliary disease, arrhythmias or vertebral fractures occurred over the median 3.3-year follow-up, a reassuring safety signal given the limited long-term tolerability data for this combination. One patient discontinued PEGV after three years due to injection site reactions.

### Impact on pegvisomant dose

Median PEGV dose was 140 mg/week, generally lower than the monotherapy doses required to achieve IGF-1 control in more resistant cases [20, 21]. We did not demonstrate a formal cumulative PEGV dose reduction at the individual patient level in our series, with dose reduction occurring in only one patient following repeat surgery. In contrast to the PAPE studies, which demonstrated cumulative PEGV dose reductions of 52 to 66% from pre-established requirements in patients already biochemically controlled on octreotide/lanreotide plus PEGV [8, 9], the lower absolute doses in our series reflect PAS additive GH suppression from the outset of combination therapy in a more refractory population.

### Tumor biology and response

The predominance of mammosomatotroph adenomas in this cohort reflects a patient population at high risk of requiring third-line treatment for control [10]. Low SSTR2 expression and a low SSTR2/SSTR5 ratio are also recognized predictors of octreotide/lanreotide resistance [3, 22, 23]. SSTR2 negativity was present in three of six patients in our series, demonstrating the predictive value of lack of SSTR2, but also that receptor profiling does not reliably identify all patients. Tumor invasiveness and degree of GH hypersecretion, seen in our case series, are likely co-determinants of resistance [10, 15]. Nonetheless, PAS-PEGV appears effective across a broader receptor landscape than the low SSTR2 paradigm alone would predict, as PAS provides additional GH suppression via SSTR5 [24, 25], and PEGV normalizing IGF-1 independently of receptor status entirely [26]. Distinguishing mammosomatotroph from mixed somatotroph-lactotroph adenomas on histopathology remains challenging even with current immunohistochemical standards, and a unified definition of GH-PRL co-secreting adenomas is still lacking [6], which may contribute to the histological heterogeneity observed across our cohort and the wider literature.

## Strength and limitations

Strengths of this series include consistent clinical management within a single tertiary Pituitary Center using a unified care protocol, standardized biochemical monitoring with the same laboratory platform across the majority of follow-up visits, and centralized pathological review of all surgical specimens regardless of the operating institution. Limitations include the small sample size and retrospective design inherent to a case series, incomplete biochemical follow-up data in some patients due to gaps in care related to insurance changes as well as a semi-quantitative nature of SSTR2 assessment by immunohistochemistry.

## Conclusion

Based on our data, we consider that combination of PAS-PEGV is a viable long-term option for treatment-resistant acromegaly, offering durable biochemical control and tumor stability including in young patients. PEGV did not significantly alter PAS-related hyperglycemia and proactive glycemic monitoring from treatment initiation is essential. This series adds real-world evidence supporting the targeted use of this combination in highly refractory disease, where prospective studies defining predictors of response are needed.

## Data Availability

No datasets were generated or analysed during the current study.
